# Research on the Application of Dynamic Process Correlation Based on Radar Data in Mine Slope Sliding Early Warning

**DOI:** 10.3390/s24154976

**Published:** 2024-07-31

**Authors:** Yuejuan Chen, Yang Liu, Yaolong Qi, Pingping Huang, Weixian Tan, Bo Yin, Xiujuan Li, Xianglei Li, Dejun Zhao

**Affiliations:** 1 College of Information Engineering, Inner Mongolia University of Technology, Hohhot 010080, China; chen_yj@imut.edu.cn (Y.C.); 20221100122@imut.edu.cn (Y.L.); hpp@imut.edu.cn (P.H.); wxtan@imut.edu.cn (W.T.); lixiujuan@imut.edu.cn (X.L.); 20221100108@imut.edu.cn (X.L.); zdj15147800894@gmail.com (D.Z.); 2 Inner Mongolia Key Laboratory of Radar Technology and Application, Hohhot 010051, China; yinbo@imut.edu.cn; 3 College of Resource and Environmental Engineering, Inner Mongolia University of Technology, Hohhot 010080, China

**Keywords:** microvariation monitoring radar, deformation time prediction, deformable body, phase noise standard deviation, dynamic course correlation

## Abstract

With the gradual expansion of mining scale in open-pit coal mines, slope safety problems are increasingly diversified and complicated. In order to reduce the potential loss caused by slope sliding and reduce the major threat to the safety of life and property of residents in the mining area, this study selected two mining areas in Xinjiang as cases and focused on the relationship between phase noise and deformation. The study predicts the specific time point of slope sliding by analyzing the dynamic history correlation tangent angle between the two. Firstly, the time series data of the micro-variation monitoring radar are used to obtain the small deformation of the study area by differential InSAR (D-InSAR), and the phase noise is extracted from the radar echo in the sequence data. Then, the volume of the deformation body is calculated by analyzing the small deformation at each time point, and the standard deviation of the phase noise is calculated accordingly. Finally, the sliding time of the deformation body is predicted by combining the tangent angle of the ratio of the volume of the deformation body to the standard deviation of the phase noise. The results show that the maximum deformation rates of the deformation bodies in the studied mining areas reach 10.1 mm/h and 6.65 mm/h, respectively, and the maximum deformation volumes are 2,619,521.74 mm^3^ and 2,503,794.206 mm^3^, respectively. The predicted landslide time is earlier than the actual landslide time, which verifies the effectiveness of the proposed method. This prediction method can effectively identify the upcoming sliding events and the characteristics of the slope, provide more accurate and reliable prediction results for the slope monitoring staff, and significantly improve the efficiency of slope monitoring and early warning.

## 1. Introduction

Slope sliding is a geological phenomenon that occurs during open-pit coal mining when slopes are subjected to water erosion, rainfall, groundwater movement, and human factors, causing them to slide in a specific direction under the influence of gravity [1]. China is rich in mineral resources, and in recent years, with the advancement of mining technology, open-pit mining activities have increased, making slope instability a major type of disaster that threatens public facilities and people’s lives and property. Early identification and warning of landslide disasters have become important tasks [2,3,4,5]. On 22 February 2023, a large-scale collapse occurred at the Xinjiang Mine in Alxa League, Inner Mongolia Autonomous Region, resulting in 53 deaths, 6 injuries, and a direct economic loss of CNY 204.3025 million. Therefore, it is crucial to pay close attention to the safety hazards of mine slopes, accurately monitor potential unstable slopes, and formulate reasonable monitoring and governance plans [6,7,8]. In 2023, the Ministry of Natural Resources issued the “14th Five-Year Plan for National Geological Disaster Prevention and Control”, proposing to establish a comprehensive prevention and control system with geological disaster risk prevention and control as the main line, enhance disaster prevention capabilities and engineering standards, and prevent and mitigate geological disaster risks. In the 2024 national geological disaster prevention and control work, it is required to fully utilize comprehensive remote sensing technology to identify and prevent major hazards [9,10].

Due to the complex nature of the rock and soil bodies of open-pit mine slopes and the various factors influencing slope instability, slopes have become one of the main safety hazards in open-pit mine engineering management activities [11]. Monitoring and managing unstable slopes have gradually become important aspects of ecological restoration in open-pit mines, and scholars at home and abroad have conducted extensive research on this [12,13,14]. In the 1960s, Saito proposed a landslide prediction model based on creep theory through landslide experiments. This model divides the landslide process into three stages, with slope instability typically occurring in the accelerated deformation stage, characterized by a significant increase in surface deformation rate [15]. Subsequently, many scholars have conducted follow-up studies based on this model, promoting the development of landslide early warning and prediction. In 1969, Hoek proposed an extension method for estimating landslide timing based on the displacement–time curve of landslide monitoring at the Chuquicamata mine in Chile [16]. In 1985, Japanese scholar Fukuzono proposed the “Fukuzono Method” for predicting the time of slope stability failure through large-scale model experiments [17].

With the development of computer technology, communication technology, systems science, nonlinear analysis theory, and intelligent technology, landslide early warning and prediction have evolved into a multidisciplinary integrated technology. This has attracted the attention of many researchers aiming to improve the accuracy of early warning and prediction, leading to the emergence of various models, methods, and systems for early warning and prediction [18]. In 2009, Qiang Xu proposed the tangent angle landslide early warning standard based on the Saito model. He found that when the tangent angle of the displacement–time curve exceeds 45 degrees, it signals that the landslide has entered the accelerated deformation stage, and when the tangent angle approaches 90 degrees, the likelihood of a landslide increases [19]. In 2020, Kamal Das and Thaljaoui et al. developed real-time threshold prediction systems for landslides based on wireless sensor networks and limit equilibrium-based landslide prediction systems, respectively. These systems have significantly improved the accuracy of predicting slope sliding time [20,21].

In recent years, with the development of remote sensing technology, ground-based micro-variation monitoring radar technology, as an important branch of InSAR technology, has represented an innovative technology in remote sensing monitoring. This technology combines the principles of synthetic aperture radar imaging and differential interferometry, enabling precise measurement of small surface changes. It has been widely applied to obtain surface deformation data over large areas, showing great monitoring potential [22,23]. Over the years, this technology, with its all-weather, all-time operation, minimal environmental interference, wide monitoring range, and high precision, has been widely applied in acquiring large-area surface deformation data [24]. The Ground-Based Synthetic Aperture Radar (GB-SAR), which evolved from this technology, has shown immense potential in geological hazard monitoring and early warning. Particularly, the development of linear, circular, and array types of GB-SAR has significantly enhanced the capability to monitor geological disasters such as landslides and ground subsidence [25]. In terms of landslide early warning and prediction, scholar Wang Yadong proposed a new method for predicting slope instability time by integrating the coherence and volume of landslide bodies with indicators of slope deformation and tangent angle [26]. The application of machine learning and neural network technology is also being extensively researched, providing important technical means for landslide monitoring [27,28,29,30]. InSAR technology itself displays significant advantages in monitoring large mining areas, particularly in the domains of surface deformation monitoring and environmental impact assessment. It provides millimeter-level precision in deformation data, covers extensive geographical areas, and reveals the dynamic processes of deformation and their potential long-term impacts through time-series analysis. Due to its cost-effectiveness and independence from ground facilities, InSAR is particularly suited for monitoring in complex or remote terrains [31,32,33]. By precisely monitoring the deformation of the surface around mining areas, InSAR not only helps evaluate the impact of mining activities on geological stability and the environment but also provides a scientific basis for mine management and environmental restoration, making it an ideal tool for managing large mining areas and environmental monitoring.

The phase information of GB-SAR is crucial data for generating high-quality radar images and performing interferometric measurements. During the SAR imaging process, the radar system emits electromagnetic pulses that are reflected back upon contact with ground targets. The phase information in the reflected echo signals reflects the distance traveled by the radar wave from transmission to reception. Since the propagation speed of electromagnetic waves is known, by precisely measuring the phase difference of the echo signals, highly accurate distance information can be calculated. In InSAR technology, the phase differences between multiple SAR images of the same area obtained at different times are used to analyze small surface changes. However, when slope deformation occurs, the interferograms of the target area are limited by linear phase models, resulting in significant noise in the generated interferogram phases [34,35,36,37]. When there is severe phase noise in local areas, the deformation information extracted from the interferograms can be inaccurate [38]. Therefore, the accuracy of phase noise in interferograms is a crucial factor affecting the magnitude of deformation values.

This paper addresses the characteristics of slope sliding timing and proposes a method to reflect the degree of slope deformation from the perspective of noise. This method is based on the dynamic process correlation between slope deformation volume and phase noise standard deviation. By combining the dynamic process correlation with the tangent angle, a comprehensive early warning strategy is formulated. Using time series data from two mining areas in Xinjiang as examples, a time prediction model is constructed, and its predictive performance is analyzed and evaluated in detail, verifying the practical application of the method.

## 2. Study Area

### 2.1. Overview of Study Area One

**Figure 1:**
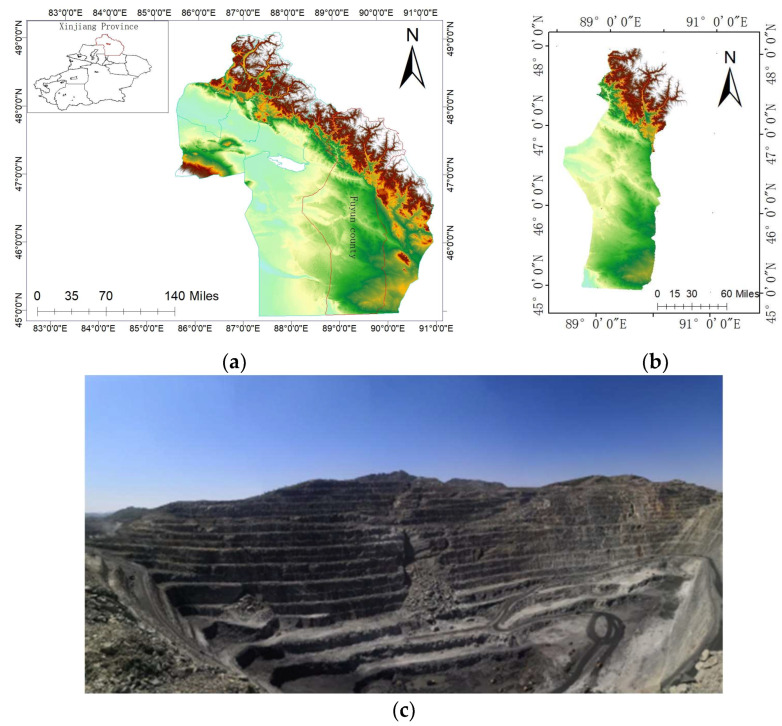
Geographic location and site image of Research Area 1: (**a**) Altay prefecture in Xinjiang Province, (**b**) Fuyun County in Altay Region, (**c**) Post-slope slide site image.

The open-pit mine in the study area is located on the slope of the middle and lower part of the Altay Mountains in northwestern China, in Altay City, Xinjiang Province. The elevation of the slope is between 1000 and 1300 m. The extent of the mine is about 1.2 km long in the northwest–southeast direction and about 0.7 km wide in the northeast–southwest direction. The terrain is characterized by a gradual increase from north to east, and the relative height difference varies by up to 50~300 m. These elevation differences are caused by the destruction of the original topography and geomorphology caused by open-pit mining activities. Therefore, a number of platforms with obvious height difference are formed, which leads to the fluctuation of terrain. The summary map of the study area and a panoramic view of the open-pit mine after the slope sliding from the radar perspective are as follows (Figure 1):

### 2.2. Overview of Study Area Two

**Figure 2:**
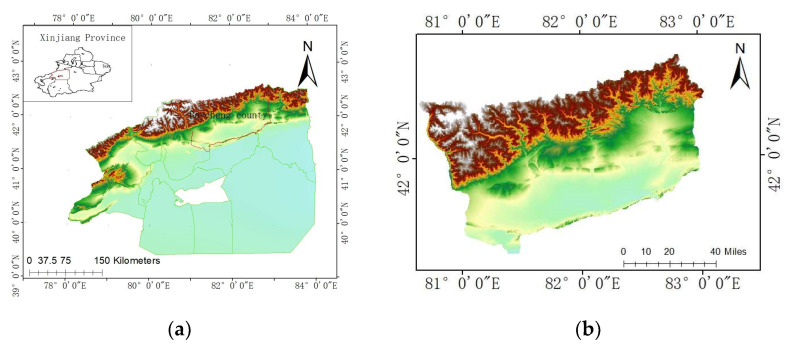
Geographic location of study area two: (**a**) Aksu prefecture in Xinjiang Province, (**b**) Baycheng county in Aksu prefecture.

Study area two is still an open-pit mine, located in the southern foot of the middle Tianshan Mountains in China, in the Aksu region of Xinjiang Province. The altitude of the coal mine is between 945 and 1020 m. The extent of the coal mine is 3.625 km long from east to west and 0.330–0.580 km wide from north to south, and the area of the mine field is about 1.527 km^2^. The summary of the geographical location of the study area is as follows (Figure 2):

In the field of geology, this study focuses on the diversity of rock types in specific areas, including five main types of gneiss, schist, granulite, marble, and amphibolite. These rocks provide key material support for the geological structure and geomorphology of the area. Especially in some slope areas, through systematic observation of rock mass, it is found that higher integrity is gradually shown from the surface to the deep layer. Although the rock mass is relatively unified, the process of crushing it inevitably leads to the development of structural fracture zones. The formation of these fault zones may be closely related to regional geological dynamics, environmental vibration factors such as seismic activity, and human excavation operations, as well as rock structure characteristics, which together affect the structural stability of rock mass. Although there are local fractures, the rocks in the whole area generally show good stability and resistance to external erosion, which effectively maintains the integrity of the geological structure and provides an important material basis for the geological safety and sustainable development of the region.

### 2.3. Experimental Equipment and Experimental Data

**Figure 3:**
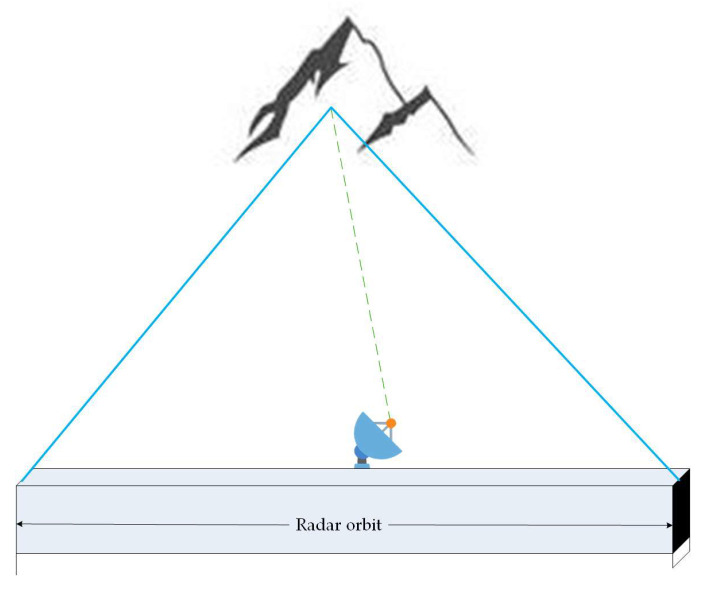
Micro-variation monitoring radar measurement diagram.

**Figure 4:**
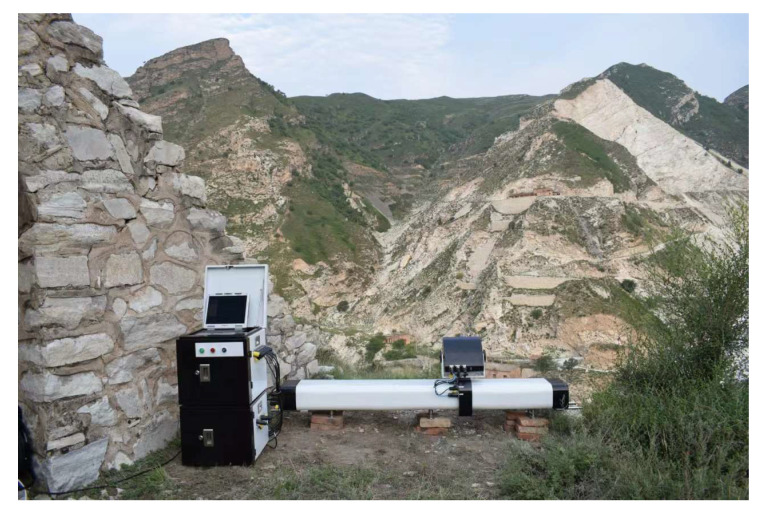
MPDMR-LSA radar system.

The two regional experiments take the synthetic aperture micro-variation monitoring radar as an example. The technology realizes the relative displacement simulation between the radar and the monitoring object based on the principle that the radar sensor moves along a predetermined linear trajectory, thereby simulating the effect of a large-aperture antenna with a small-aperture actual antenna. In the distance dimension, this method achieves high-resolution distance measurement by transmitting and receiving electromagnetic wave signals and using pulse compression technology. In the azimuth dimension, the electromagnetic wave signal is processed by coherent accumulation through the motion of the sensor along the straight-line trajectory to achieve high azimuth resolution. Thus, a high-resolution two-dimensional image of the monitoring area is generated. The working principle of the radar is shown in Figure 3:

The micro-variation monitoring radar used in the two experiments is the MPDMR-LSA system developed by Inner Mongolia University of Technology (Figure 4), and the system parameters are shown in Table 1.

**Figure 5:**
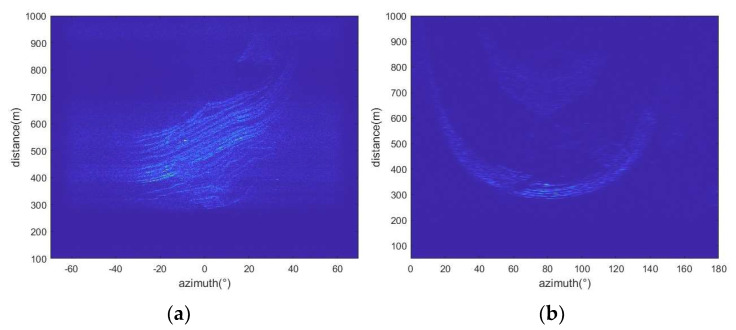
Radar slices of two research areas: (**a**) Study area one, (**b**) Study area two.

The data come from two research areas in different time periods. The monitoring time of the first research area began on 18 April 2021 and ended on the morning of 21 April 2021. A total of 225 radar images were obtained. The monitoring time of study area two began on 11 September 2023 and ended on the morning of 12 September 2023. A total of 126 radar images were obtained. Figure 5 shows the radar images of the two research areas obtained by the micro-variation monitoring radar. Since the monitoring environment is mainly composed of rock structures with strong scattering performance, the obtained imaging results show high clarity.

## 3. Methods

**Figure 6:**
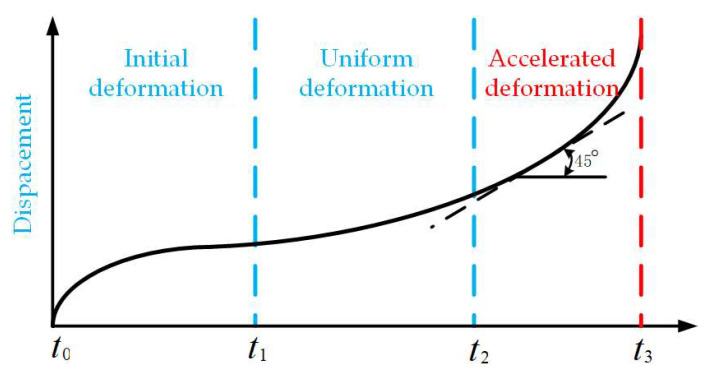
Saito curve model and tangent angle model.

**Figure 7:**
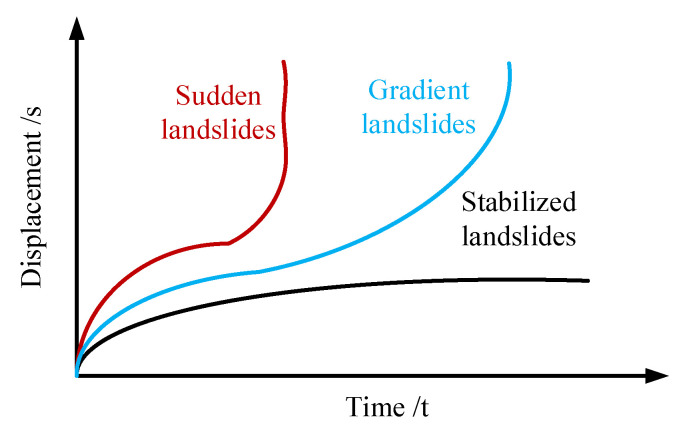
Three types of displacement–time curves of slope deformation.

The development process of a landslide usually occurs over an obvious time span, during which the cumulative displacement and time relationship show three stages of characteristic changes. These three stages are: the initial deformation stage, in which the deformation development is slow; subsequently, the landslide enters the stage of uniform deformation, and the displacement rate of the landslide remains relatively constant. Finally, it enters the accelerated deformation stage. At this stage, the deformation rate of the landslide increases rapidly, resulting in the final failure, as shown in Figure 6. In this process, the landslide will show significant macroscopic deformation and failure characteristics. Especially in the stage of deformation acceleration, there is usually pre-landslide precursor information, which is very important for the advanced prediction and warning of a landslide. Based on these observations, the early detection and emergency warning system for landslides is realized, which provides a scientific basis for the risk management and mitigation strategy of landslide disasters. Through in-depth study of the deformation behavior and failure mechanism of various types of landslides, and a comprehensive analysis of the morphological characteristics of a large number of monitored displacement–time curves, it is concluded that under different stress conditions, different types of landslide displacement–time curves can be divided into a set of gradient deformation curve groups, as shown in Figure 7. These curve groups can be classified into three main deformation modes: gradual, sudden and stable.

As a tool for monitoring landslide activities, the displacement–time curve has significant advantages in providing early warning, but its ability is limited when dealing with sudden landslides, especially in providing sufficient early warning time for such landslides. In addition, a single displacement–time curve mainly provides quantitative data of slope displacement, and these data may not reveal the complete potential risk of landslides without other context information. Therefore, this study proposes a comprehensive method to enhance the accuracy and effectiveness of landslide warning through the combination of multiple parameters.

### 3.1. Forecasting Process

**Figure 8:**
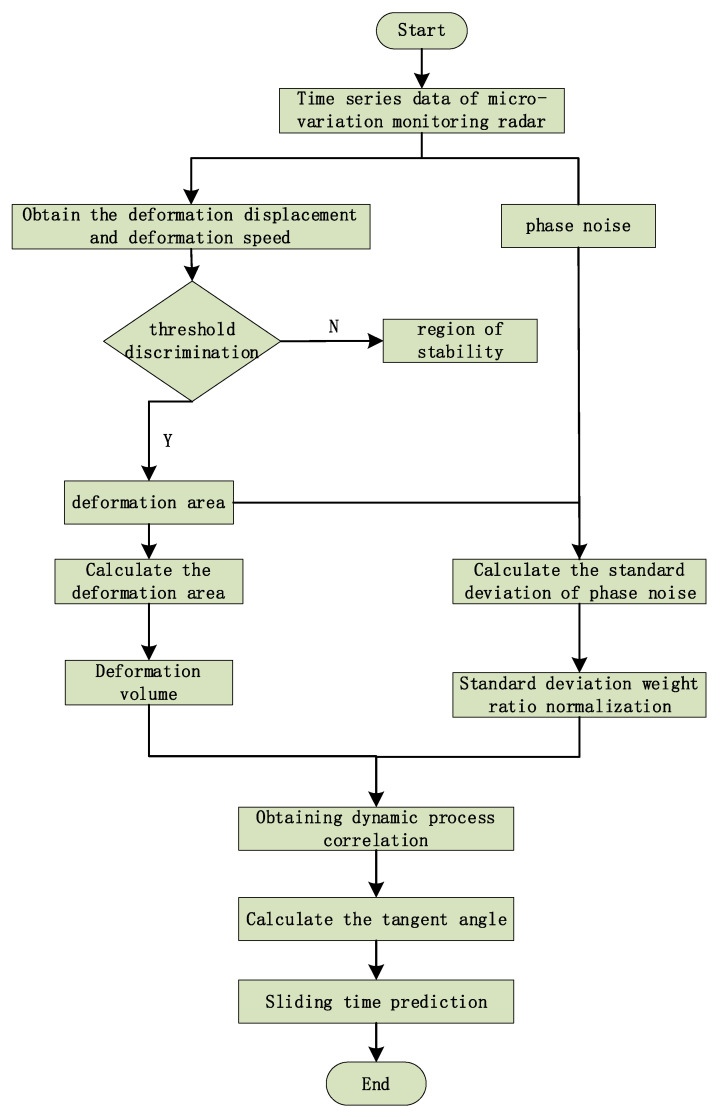
Slope slide early warning flowchart.

The slope sliding time prediction method based on the dynamic history correlation of the volume of the deformation body and the standard deviation of the phase noise combined with the tangent angle mainly includes five parts, namely, the acquisition of the displacement of the slope deformation body, the extraction of the volume of the deformation body, the normalization of the standard deviation of the phase noise, the calculation of the dynamic history correlation, and the combination of the tangent angle model. The overall process is shown in Figure 8:

In the figure, the threshold includes the slope displacement threshold and the slope velocity threshold; the threshold is that the displacement and velocity of the deformation body at a certain moment are greater than the slope displacement threshold and velocity threshold.

### 3.2. Acquisition of Displacement of Slope Deformation Body

**Figure 9:**
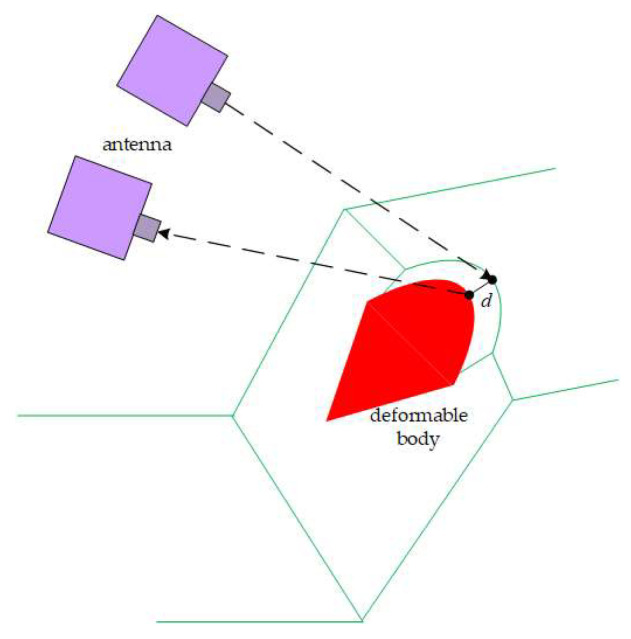
Working principle of deformation monitoring with micro-variation monitoring radar.

In the micro-variation monitoring radar, after the accurate registration of the radar single-look complex image (SLC) at different times in the same area, the displacement in the radar line-of-sight direction can be obtained by calculating the phase change of the target point in the two phases. The principle of interferometry is shown in Figure 9.

It is assumed that the initial complex image observed by the micro-variation monitoring radar is $I_{1}$. The complex image of the moment after the change is $I_{2}$; these can be expressed as:

(1) $$I_{1}=|I_{1}|e^{i\varphi_{1}}$$

(2) $$I_{2}=|I_{2}|e^{i\varphi_{2}}$$

The phase difference between two complex images of continuous time series can be used to calculate the displacement of the target point between the two observations. The small deformation obtained by differential interferometry also needs to be filtered to obtain the true deformation value. The phase difference calculation formula is

(3) $$T_{i}^{s}=-\frac{\lambda }{4\pi }(\varphi_{2}-\varphi_{1})$$

In the formula, $T_{i}^{s}$ is the deformation of the target point between two observations. $\varphi_{1}$ and $\varphi_{2}$ are the phases of the two observations, respectively, and λ is the wavelength of the radar wave.

In the process of long-term geological monitoring, random factors such as human activity interference, local vibration caused by construction, and noise of the monitoring system itself may cause fluctuations in monitoring data. If only relying on the change of a single feature pixel for region recognition, the fluctuation of pixel values caused by these interferences may be mistaken for pixel distortion. However, geological disasters such as landslides usually involve significant deformation over a wide range, rather than limited changes in a few isolated feature pixels. Therefore, this study proposes a feature pixel selection strategy based on deformation velocity threshold (v) and cumulative displacement threshold (s). The strategy aggregates the detected feature pixels through the connectivity algorithm to identify the sensitive areas that need to be preferentially monitored.

### 3.3. Extraction of Slope Deformation Volume

In the application of micro-variation monitoring radar, because the transmission and reception of a radar beam are completed at different times, the performance of azimuth resolution is represented by azimuth angle, which leads to the heterogeneity of the surface area distribution represented by each pixel in radar data. Specifically, in the area near the radar station, the actual surface area covered by the corresponding pixels is relatively small; on the contrary, as the distance from the radar station increases, the actual surface area covered by the corresponding pixel gradually increases. This phenomenon causes the projection of each radar pixel on the surface to appear as a fan-shaped annular region. The area of the fan-shaped annular region can be accurately calculated and determined by the key parameters of the radar, including the range resolution, azimuth resolution, and the distance from the radar to the target [39]. The calculation formula is as follows:

(4) $$S^{n}=\frac{2n-1}{2}\alpha \beta^{2}$$

In the formula, $S^{n}$ is the area of a single pixel; α is azimuth resolution; β is the distance resolution; n is the sampling position of the corresponding pixel in the distance direction.

In the study of mine slope stability, the volume change of the deformation body is the key factor to predict and prevent slope sliding. The volume change reflects the dynamic adjustment of the internal structure and density of the slope, which is closely related to the displacement vector of the slope and its change rate. The volume increase or decrease caused by the development of cracks or the change of material density in the slope may indicate a significant change in slope stability. This is because the volume adjustment directly affects the mechanical balance and stress distribution of the slope, which determines the potential risk of slope sliding. Therefore, accurate monitoring and analysis of the volume change of slope deformation is a key step in formulating effective preventive measures and intervening in slope instability. The calculation method of slope volume is as follows:

(5) $$V_{i}=(\sum_{n=1}^{N}(D_{i}^{n}\times S^{n}))/N$$

In the formula, $V_{i}$ is the deformation volume of the i moment; $D_{i}^{n}$ is the deformation value of the n pixel after filtering at the i moment; N is the total number of pixels in the sensitive area. Due to the gradual deformation of the slope, its range is gradually expanding, so the number of pixels in the sensitive area is gradually increasing.

The method introduced in this paper involves a mathematical model for estimating the overall slope volume by combining the calculations of the volumes of all parts of the slope. In the slope sliding warning system, the estimation of the slope volume is very important, because it directly affects the assessment of the potential threat of the slope and determines the scale and influence range of the slope. Larger slope sliding will cover a wider area and cause more serious damage. Accurate assessment of the volume of slope sliding can improve the frequency of emergency response and reduce casualties and property losses. The volume calculation model formula (5) described in this paper indicates that the slope volume is approximately calculated by the sum of the volumes of the sections.

### 3.4. Normalize the Standard Deviation of Phase Noise

In the field of micro-variation monitoring technology, phase noise is the main factor affecting the accuracy of deformation measurement. Its intensity directly affects the accuracy of elevation measurement, the effectiveness of deformation monitoring, and the integrity of phase information. The phase noise is mainly caused by the noise in the system, the baseline decoherence effect, and the error caused by the time decoherence effect. These factors may not only lead to a significant decrease in the accuracy of elevation measurement, but may also weaken the ability of deformation monitoring. In synthetic aperture radar (SAR) imaging technology, the coherence coefficient between two SAR images used to generate interferograms has a direct impact on the phase noise level. Furthermore, the degree of phase noise can be quantified by the coherence coefficients of these two SAR images. The larger the coherence coefficient, the smaller the phase noise. Franceschetti et al. gave the relationship between the standard deviation of phase noise and the coherence coefficient in this paper:

(6) $$\sigma_{i}^{n}=\int_{-x}^{x}{\left(\varphi -\varphi_{0}^{n},j\right)}^{2}\rho (\varphi ,\gamma_{i}^{n},1,\varphi_{0}^{n},j)d\varphi$$

In the formula, $\sigma_{i}^{n}$ is the standard deviation of the phase noise of the n pixel at the i moment; φ is the interference phase; $\varphi_{0,i}^{n}$ is the initial phase of the n pixel at the i moment. $r_{i}^{n}$ is the corresponding coherence coefficient.

$\rho (\varphi ,\gamma_{i}^{n},1,\varphi_{0,i}^{n})$ represents the phase probability density function with the multiplicity of 1, and its analytical expression is as follows:

(7) $$\begin{array}{c} \rho (\varphi ,\gamma_{i}^{n},1,\varphi_{0}^{n},)=\frac{\Gamma (1.5)(1-{\left(\gamma_{i}^{n}\right)}^{2})\beta }{2\sqrt{\pi }\Gamma (1){\left(1-\beta^{2}\right)}^{1.5}}+\frac{1-\beta^{2}}{2\pi }\cdot \\ F(1,1;1/2;\beta^{2}),-\pi <(\varphi -\varphi_{0}^{n})<\pi \end{array}$$

In the formula, Γ(g) is the gamma function; $\beta =\gamma_{i}^{n}(\varphi -\varphi_{0,i}^{n})$; F(g) represents the Gaussian hypergeometric function.

The coherence coefficient mentioned in the above formula measures the similarity of the radar echoes of the same surface points observed at different time points, that is, the similarity of the observation results of the target at two different times. The phase consistency of the observed target will gradually decrease with the change between the two radar scans. When the coherence coefficient is 0 in the ideal state, the target is completely incoherent. The coherence coefficients of the two SAR images are calculated as follows:

(8) $$\gamma_{i}^{n}=\frac{\left|\sum_{n\in W}S_{i}(n)\times \bar{S_{i+1}(n)}\right|}{\sqrt{\sum_{n\in W}{\left|S_{i}(n)\right|}^{2}\times \sum_{n\in W}{\left|S_{i+1}(n)\right|}^{2}}}$$

In the formula, $S_{i}(n)$ and $S_{i+1}(n)$ are the n echo values of the i and i+1 time in the window W respectively; $\bar{S_{i+1}(n)}$ denotes the conjugate complex number of $S_{i+1}(n)$; $r_{i}^{n}$ is the calculated nth coherence coefficient at time i.

Due to the large data fluctuation of the phase noise standard deviation at different positions at the same time, the average of the phase noise standard deviation can improve the stability of the calculation process, ensure that all the phase noise standard deviation is equally important, and avoid the calculation results dominated by the prominent characteristics of some values. The average standard deviation of phase noise is calculated as follows:

(9) $$\sigma_{i}=(\sum_{n=1}^{N}\sigma_{i}^{n})/N$$

In the formula, $\sigma_{i}$ is the average standard deviation of phase noise; N is the total number of pixels in the sensitive area.

### 3.5. The Calculation of the Dynamic Process Correlation and Its Fusion with the Tangent Angle Model

Conventionally, landslide monitoring and prediction often rely on single-pixel analysis methods, which play a key role in analyzing surface deformation and predicting sliding. However, there are also some limitations. For example, single-pixel analysis will be disturbed by various environmental factors, atmospheric disturbances, and other seasonal changes. These factors will have a significant impact on the monitoring results, resulting in misjudgment of slope stability. Therefore, from the overall point of view, this paper proposes the dynamic history coherence between the volume of the deformation body and the average standard deviation of the phase noise. In order to describe the deformation degree of the slope over a period of time, the calculation is as follows:

(10) $$DCC=\frac{V_{i}}{\sigma_{i}}$$

where DCC is the dynamic history coherence between the volume of the deformation body and the average standard deviation of the phase noise.

In this study, an innovative slope sliding time prediction technology is introduced, which combines multi-threshold judgment and dynamic history coherence tangent angle technology. When calculating the coherence of the dynamic process, the overall volume of the deformation body is comprehensively considered, and the phase noise is evaluated. In the face of the complexity of slope sliding and the difficulty of in-depth analysis of complex data obtained by micro-variation monitoring radar, an improved method of tangent angle of dynamic process coherence of slope sliding is proposed to improve the depth and accuracy of data analysis.

When monitoring a specific area to predict the occurrence of landslides, the comprehensive multi-threshold and dynamic process coherence tangent angle technology can effectively identify the landslide risk in the terrain acceleration stage. This method can not only make accurate prediction before landslide events, but also provide an effective technical scheme for a landslide early warning system in open-pit mines. In the process of analyzing the gradually deformed mine slope, it is observed that with the accumulation of deformation, the measurement value of dynamic history correlation gradually increases. After reaching the preset threshold, the possibility of landslide occurrence will increase significantly. Usually, before the landslide approaches, the curve of dynamic process correlation will show a significant upward trend, which provides key data support for taking preventive measures in time.

## 4. Results and Analysis

In order to verify the effectiveness and relative advantages of the slope sliding time prediction method proposed in this study, this paper selects two slopes in the Xinjiang mining area as case study objects. By using the prediction method proposed in this study and combining it with a large number of micro-variation monitoring data, a time prediction model is constructed. Subsequently, the prediction performance of the model in the actual situation is analyzed and evaluated in depth.

**Figure 10:**
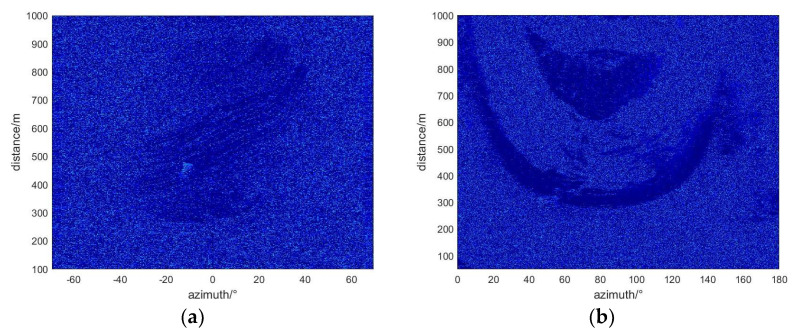
Interferogram of the study area: (**a**) Study area one, (**b**) Study area two.

**Figure 11:**
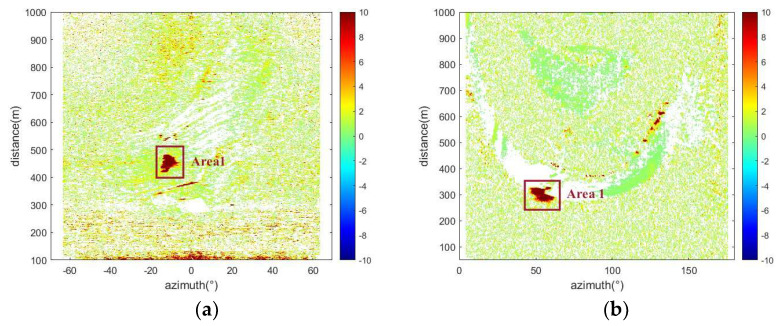
Cumulative displacement deformation: (**a**) Study area one, (**b**) Study area two.

**Figure 12:**
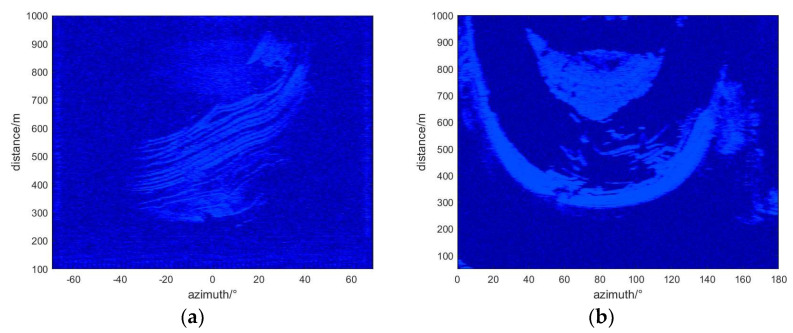
Coherence map of the study area: (**a**) Study area one, (**b**) Study area two.

**Figure 13:**
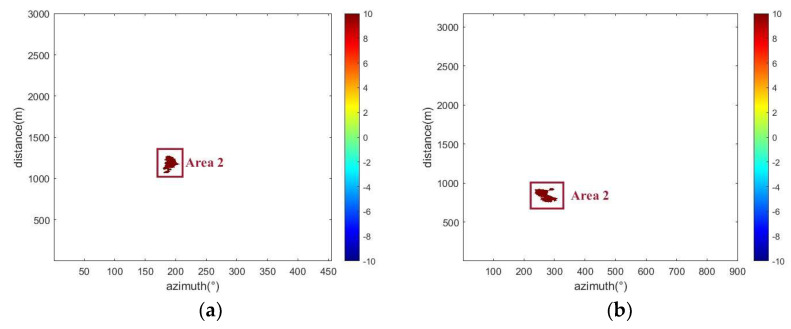
The deformed pixels are finally selected: (**a**) Study area one, (**b**) Study area two.

In this study, a predictive model was applied to process differential interferometric images from radar data collected from two mining areas during the time windows of 18 April to 21 April 2021, and 11 September to 12 September 2023. This processing yielded geological differential interferograms of the two monitoring areas (see Figure 10). Detailed analysis of cumulative deformation displacement in specific areas was conducted, with Figure 11 displaying the cumulative deformation situations in the two mining areas during the corresponding time periods and highlighting significant changes in pixel deformation values. These results emphasized significant surface deformations observed during the monitoring period, providing key evidence for further study. Additionally, it was found that the coherence in the deformation zones of slopes changed significantly when deformation occurred, as shown in Figure 12, which displays the coherence changes in the two study areas. To more precisely identify and analyze surface deformations, this paper used a conditional threshold selection method and connected the selected images after processing, significantly enhancing the accuracy and reliability of the data analysis. Figure 13 presents the post-selection results, clearly showing that areas 1 and 2 in the two mining areas are positioned similarly within the observation scene. Particularly in area 2, most pixels were successfully identified and filtered out, indicating that this area experienced significant surface deformation activity during the observation period.

**Figure 14:**
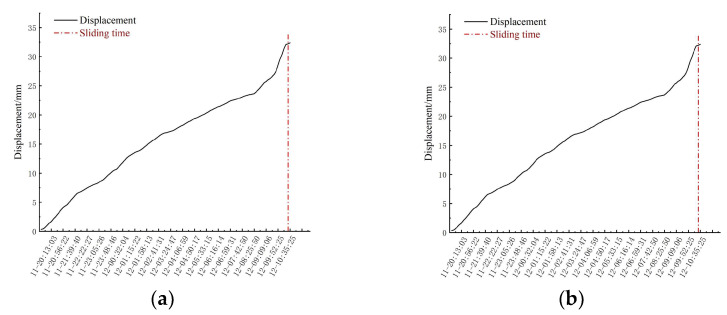
Cumulative displacement curve: (**a**) Study area one, (**b**) Study area two.

By extracting the deformation of the selected area, the displacement of the study area shows a continuous increasing trend (Figure 14), but the cumulative displacement curve of study area one basically conforms to the ‘three stages’ of the Saito model landslide, and the cumulative displacement curve of study area two basically conforms to the uniform acceleration stage and the variable acceleration stage. The displacement and calculated velocity extracted at different times in the landslide area are shown in Table 2.

It can be seen from Figure 12 that the two monitoring areas show different changes. From 02:27 on 18 April to 02:00 on 19 April, the monitoring area showed a relatively stable state, and the displacement increased slightly, but there was no obvious geological deformation. Then, in the later period, that is, from 03:00 on 19 April to 19:00 on 20 April, the geological deformation of the monitoring area entered the stage of constant velocity deformation, and the deformation was relatively stable. From 19:00 on 20 April to about 7:50 on 21 April, the geological deformation of region 1 experienced an accelerated deformation stage. The displacement of monitoring area 2 shows an increasing trend from 19:00 on 11 September to 02:00 on 12 September, and an obvious deformation stage begins to appear. Then, from 03:00 on 12 September to 08:00 on 12 September, it shows a constant speed deformation stage. At this time, although the deformation speed fluctuated slightly, the overall deformation was relatively stable. Subsequently, the region began to undergo an accelerated deformation stage. Table 2 shows the cumulative displacement of the whole process of deformation. In the accelerated deformation stage, the cumulative displacement increases sharply, and the deformation speed also shows an increasing trend. The specific numerical results show that the maximum cumulative displacement of the study area reached 56.98 mm, and the maximum speed reached 10.1 mm/h; the maximum cumulative displacement of study area two reached 32.38 mm, and the maximum speed reached 5.98 mm/h.

**Figure 15:**
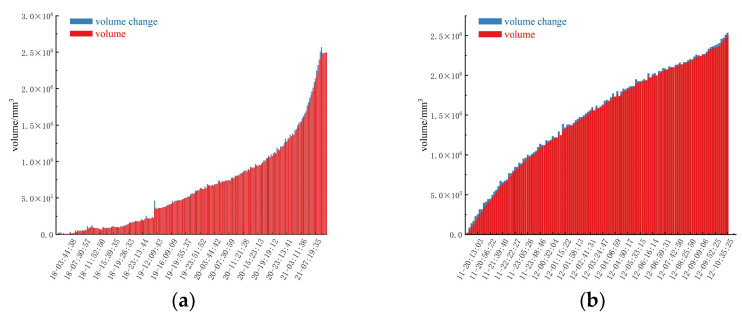
Cumulative volume change: (**a**) Study area one, (**b**) Study area two.

**Figure 16:**
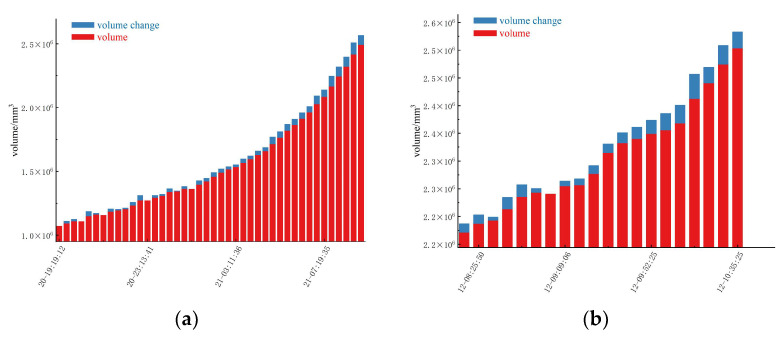
Volume change during the acceleration deformation stage: (**a**) Study area one, (**b**) Study area two.

In the research and practice of slope sliding warning, the slope volume calculation after identifying the research area of the slope is a key factor, and its accuracy is directly related to the potential hazards of the slope and the possible impact range. The volume of the two study areas’ changes over time are shown in Figure 15:

It can be seen from the figure that the volume change is displayed in two different sequences: blue represents the volume change, and red represents the volume of the deformation. In the initial stage, the volume change of study area one is relatively small and stable, which indicates that the deformation of the slope is maintained at a safe level at the initial stage, and the increase in the volume of the deformation body is not significant. On the contrary, the volume of study area two shows a large change from the beginning. With the passage of time, especially in the accelerated deformation stage of the slope (Figure 16), the slope volume shows a significant growth trend, while the growth trend of the variation of the slope deformation volume gradually becomes larger. The sharp increase in the deformation volume of the slope may indicate that after a certain point in time, the stability of the slope decreases significantly, resulting in a rapid increase in the deformation volume. In addition, a sharp increase in the volume of deformation at the end may indicate a critical state [40], which may lead to slope instability or collapse.

**Figure 17:**
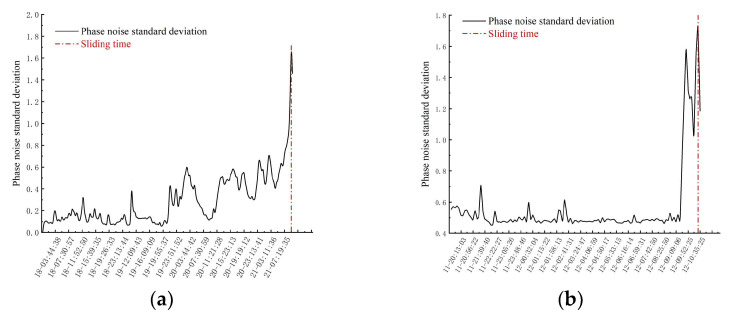
Phase noise standard deviation curve during the deformation stage: (**a**) Study area one, (**b**) Study area two.

Before the obvious sliding of the slope, the displacement curve of the slope shows a horizontal trend, and the speed shows a trend of small up-and-down fluctuation (Table 2). This is due to noise and other reasons, such that the speed cannot show a stable trend. When the slope begins to deform rapidly, the displacement begins to rise rapidly, the speed increases rapidly, and the standard deviation of phase noise at the same position increases rapidly (Figure 17).

In study area one, the data on the morning of 20 April showed that the standard deviation of phase noise gradually increased, and increased significantly at 7:00 on 21 April, revealing the overall trend of phase noise. In contrast, the standard deviation of phase noise in study area two remained stable before 6:00 on 9 September, but then began to increase significantly (Figure 17). The variation in the standard deviation of the phase noise provides a new prediction index, which is helpful for the optimization of the monitoring and slope warning system.

**Figure 18:**
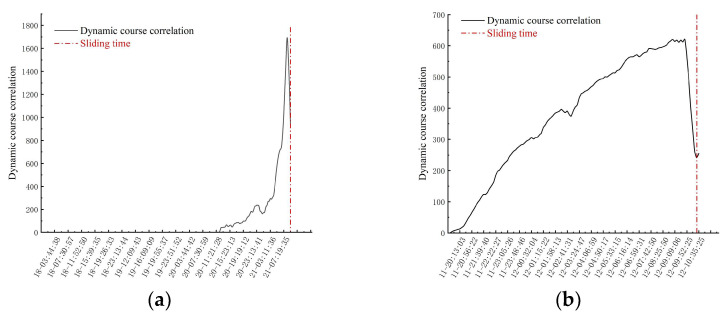
Dynamic process correlation curve of the deformation area: (**a**) Study area one, (**b**) Study area two.

By incorporating the dynamic phase noise standard deviation into the calculation (Figure 18), not only can the deformation trend of the landslide area be simulated more accurately, but also, the dynamic history correlation curve shows more concise and intuitive features than the velocity change curve. It is worth noting that the starting point of the rise of the dynamic process correlation curve closely corresponds to the accelerated deformation stage defined in the Saito model, which further verifies its application value.

**Figure 19:**
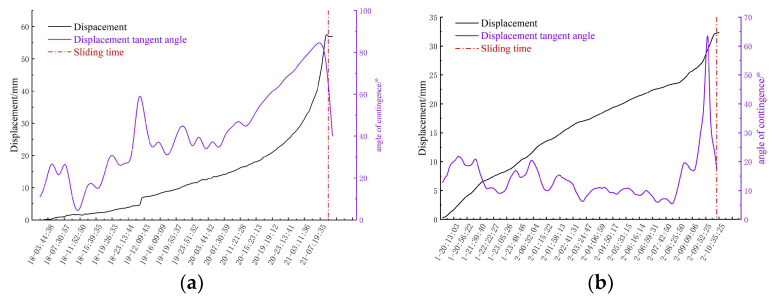
Tangent angle of displacement in the deformation area: (**a**) Study area one, (**b**) Study area two.

On the eve of the critical sliding of the slope, the correlation of the dynamic process shows a sharp decline, and reaches the lowest value when the landslide occurs. With the gradual recovery of regional stability after the landslide, the standard deviation of phase noise gradually decreases. The comprehensive analysis shows that the dynamic history correlation curve can effectively reflect the overall development trend of the slope in its accelerated deformation stage. Accuracy is a crucial step in the process of identifying the uniform deformation stage in the displacement–time curve. The correct identification of this stage is affected by many factors, including environmental factors such as temperature and rainfall, as well as human factors, which may lead to rapid or slow fluctuations in the deformation rate during the constant velocity deformation stage. This fluctuation increases the complexity of determining the deformation rate in the constant velocity deformation stage, which requires dynamic adjustment according to the actual deformation situation (Figure 19).

**Figure 20:**
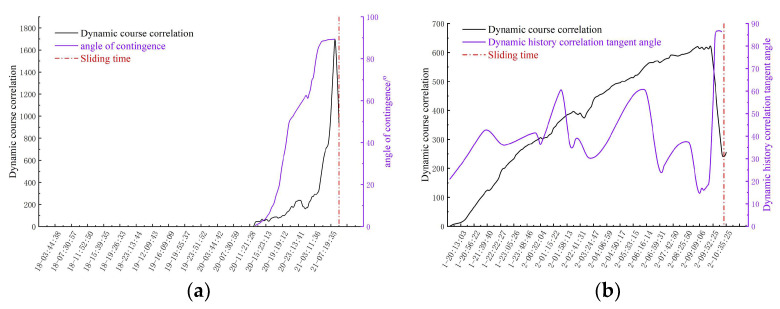
Tangent angle of dynamic process correlation in the deformation area: (**a**) Study area one, (**b**) Study area two.

In this study, the dynamic history correlation analysis was initiated by observing the case where the thresholds of cumulative displacement and deformation velocity were exceeded. After this threshold, the dynamic history correlation curve shows obvious slope fluctuations in a short time scale. This fluctuation highlights the important changes in the dynamic response at the critical stage and provides a key time window for in-depth understanding of landslide activity. Compared with the displacement velocity, the observation window of the history correlation curve in the homogeneous deformation stage is more limited, thus simplifying the determination process of the homogeneous deformation rate. As shown in Figure 20, the tangent angle curve of the dynamic history correlation clearly and intuitively shows the trend change, and its maximum tangent angle value usually appears at the moment of landslide occurrence.

In this study, by integrating a predictive algorithm of volume and phase noise standard deviations with a tangent angle model, we successfully predicted the expected occurrence times of slope sliding. Empirical analysis indicated that in study area one, on 21 April 2021, at 7:05 AM, monitoring equipment recorded a deformation rate of 9.77 mm/h and a cumulative displacement of 45.09 mm. Simultaneously, the dynamic correlation tangent angle rose to the threshold, triggering the highest landslide alert. In study area two, on 12 September 2023, at 10:06 AM, monitoring equipment recorded a deformation rate of 6.17 mm/h and a cumulative displacement of 30.37 mm. At this time, the dynamic process correlation tangent angle rose to 89.1 degrees, triggering the highest alert. Analysis of the displacement tangent angle shows that once a slope enters the slipping process and the tangent angle exceeds a certain threshold, a landslide event is imminent and enters an irreversible phase, with this moment being the critical time. According to regulations, once the dynamic process correlation tangent angle reaches the alert threshold, the highest warning should be issued at least two hours in advance to ensure the safety of the mining area. Through this warning mechanism, timely evacuation of mining facilities was successfully executed in both events, significantly reducing the risk of property and personnel losses.

However, it should be noted that despite the overall increasing trend in the tangent angle curve, the fluctuation of the monitoring data may still lead to misjudgment. Therefore, in the application of this prediction method, the fine analysis and in-depth interpretation of the data is the key to ensure the accuracy and reliability of the monitoring results. Through specific case analysis, this study verifies the effectiveness and practicability of the landslide early warning mechanism and shows the important role of scientific monitoring and early warning in disaster management.

## 5. Discussion

Traditionally, early warning systems often issue early warning signals by setting the threshold of a single pixel. However, this method has a significant flaw: a single pixel is susceptible to environmental changes, such that monitoring data may fluctuate suddenly or continue to increase, resulting in false positives. Therefore, it is less reliable to judge the risk of landslides by relying on the data change of single pixel point. In order to solve this problem, modern technology has begun to adopt remote sensing monitoring technology and apply point cloud data for analysis, so as to avoid the limitations of relying on a single data point. Although this new method improves the overall reliability of early warning, environmental and human factors may still introduce noise and affect the accuracy of monitoring. Therefore, this study proposes a new dynamic history threshold warning index for landslide monitoring and related phase noise factors, which combines the analysis of deformation volume and phase noise. In addition, by integrating the tangent angle model, this paper further proposes a comprehensive early warning system suitable for open-pit mine slope landslides.

In the field of geological monitoring, determining the appropriate threshold for slope deformation has always been a key challenge, and there is currently no widely accepted method for effective threshold determination. Typically, threshold determination is based on the analysis of long-term cumulative data, and as more data accumulate, these thresholds need continuous optimization and adjustment to more accurately reflect actual geological conditions. The setting of thresholds is influenced by various factors, including the size of the slope, the characteristics of the strata, and the types of rock and soil. In practical monitoring operations, historical data analysis methods are commonly used, involving the evaluation of the maximum deformation rates observed in the past and using these as the unacceptable maximum deformation rate thresholds during landslide events. When determining specific thresholds, it is essential to consider the properties of the geotechnical body, the degree of human interference, the potential impact area, and other factors that may lead to an increase of 10–20% in the maximum deformation rate. If the monitored slope deformation rate exceeds this threshold, the stability of the slope needs to be closely monitored. If no landslides or signs of landslides are observed, the threshold should be adjusted and updated based on the current deformation rate. Although the method proposed in this study has been validated in two mining areas, we recognize that these data may not be sufficient to comprehensively demonstrate its applicability across all mining sites. These two mining areas represent only a limited number of soil types. Therefore, future research needs to test this method in more diverse mining areas and soil types to further verify its effectiveness and reliability in different environments. This will help to more comprehensively evaluate the method’s potential for landslide prediction and enhance its generalizability.

Based on long-term monitoring experience, considering the influence of noise on deformation monitoring and the nonlinear inverse relationship between deformation and coherence, this study proposes an innovative angle-cut warning criterion. In the process of interferogram inversion deformation, the influence of noise cannot be ignored, which will significantly affect the accuracy of deformation data. In addition, the deformation process of the target area itself will also change its scattering characteristics, and weather factors, such as rainfall and snowfall, may also affect the echo characteristics of the radar signal, thus affecting the accuracy of the inversion results. Therefore, ensuring the high accuracy of deformation data is a key prerequisite for accurate early warning. It should be noted that phase noise is an inevitable persistence factor. Only when the deformation velocity and cumulative displacement reach a certain degree can the dynamic history correlation be effectively calculated. At the same time, the accurate identification of the landslide acceleration stage depends on the accumulation and in-depth analysis of a large amount of data.

## 6. Conclusions

(1) This study conducted a comprehensive analysis of the relationship between slope sliding time and the response of the sliding body, considering key factors such as tangent angles, sliding volume, and phase noise. This analysis led to the development of a model that precisely predicts the timing of slope sliding. To validate the effectiveness of the model, two mining areas in Xinjiang were selected as case study sites. The experimental results demonstrate that the model can effectively predict the pre-sliding time of slopes, and the prediction usually occurs earlier than the actual sliding events, thereby confirming the model’s strong predictive capability and its practicality in real-world engineering applications.
(2) Drawing on long-term monitoring experience, this study considered the nonlinear inverse relationship between phase noise and coherence, and integrated this model with a tangent angle model to propose a dynamic process-related tangent angle criterion. By precisely analyzing the tangent angle of the model, this method significantly improved the accuracy and reliability of the early warning system. To verify the effectiveness of the proposed warning criterion, experimental validation was conducted in two mining areas. The experimental results showed that when the tangent angle reaches a specific threshold, slope collapse occurs, thereby confirming the practical value and effectiveness of the warning criterion in real applications.
(3) Based on the comprehensive analysis above, this study has successfully developed and validated a model for predicting the timing of slope sliding. Through precise data monitoring, the model significantly enhances the accuracy of monitoring data and improves the practicality and effectiveness of the early warning system. Utilizing this innovative technology and clear warning criteria, the method can more effectively predict and prevent potential geological disasters, thereby providing robust technological support for mine area safety management. These achievements not only enhance the application efficiency of existing technologies but also contribute significant academic value to the field of geological disaster prevention.

## Figures and Tables

**Table 1:** Parameters of micro-variability monitoring radar.

Parameter	Numerical Value
Frequency range	16.5 GHz~17.5 GHz
Monitoring range	60° × 30°
Picture resolution	0.3 m × 0.0054 rad
Monitoring accuracy	0.1 mm
Operating distance	5 km
Working temperature	−30 °C~50 °C

**Table 2:** Parameters of slope sliding: (a) Study area one, (b) Study area two.

Time	s/mm	v/(mm·h^−1^)	Time	s/mm	v/(mm·h^−1^)
(a)					
18-02:27:19	0	0	19-23:51:52	11.41	0.64
18-05:00:03	0.61	0.35	20-02:22:38	12.46	0.09
18-07:30:57	1.05	0.18	20-05:00:06	13.59	1.16
18-10:18:57	1.66	0.02	20-07:30:59	14.13	0.11
18-13:08:24	1.89	0.15	20-10:05:56	15.30	0.29
18-15:39:35	2.16	0.07	20-12:37:00	16.64	0.40
18-18:10:51	2.50	0.25	20-15:23:13	17.93	0.24
18-20:42:16	3.34	0.24	20-17:54:28	19.91	0.27
18-23:13:44	3.92	0.26	20-20:34:53	22.39	2.01
19-01:44:45	4.57	0.54	20-23:13:41	25.05	3.91
19-13:31:00	7.32	0.06	21-01:48:20	28.65	6.41
19-16:09:09	8.24	0.42	21-04:29:47	34.07	10.01
19-18:40:06	9.02	0.08	21-07:19:35	45.09	9.77
19-21:02:43	10.17	0.85	21-09:50:36	56.98	0
(b)					
11-19:36:58	0.35	0	12-04:14:12	18.41	1.52
11-20:41:55	3.28	3.35	12-05:18:49	19.98	1.41
11-21:46:22	6.53	3.05	12-06:23:27	21.50	1.59
11-22:51:19	8.12	1.60	12-07:28:24	22.80	1.09
11-23:55:59	10.38	2.46	12-08:33:03	23.97	1.10
12-01:00:56	13.12	2.58	12-09:37:58	26.98	2.86
12-02:05:26	15.10	2.30	12-10:06:32	30.37	6.17
12-03:10:21	16.95	1.62	12-10:35:25	32.38	5.89

## Data Availability

The data presented in this study are available on request from the corresponding author. The data are not publicly available due to privacy.
